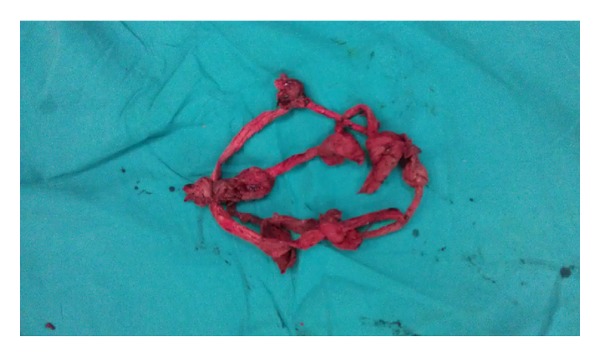# The Uterine Sandwich Method for Placenta Previa Accreta in Mullerian Anomaly: Combining the B-Lynch Compression Suture and an Intrauterine Gauze Tampon

**DOI:** 10.1155/2013/236069

**Published:** 2013-03-31

**Authors:** Mustafa Kaplanoğlu

**Affiliations:** Hatay State Hospital Obstetric and Gynecology Department, 31030 Antakya, Turkey

## Abstract

Mullerian duct anomalies may cause obstetric complications, such as postpartum hemorrhage (PPH) and placental adhesion anomalies. Uterine compression suture may be useful for controlling PPH (especially atony). In recent studies, uterine compression sutures have been used in placenta accreta. We report a case of PPH, a placenta accreta accompanying a large septae, treated with B-Lynch suture and intrauterine gauze tampon.

## 1. Introduction

Postpartum hemorrhage (PPH) is a significant factor in maternal mortality and morbidity. Placental abnormalities are a major contributor to obstetric hemorrhage. The common placental abnormalities include placental abruption, placenta previa, and adherent (accreta, increta, percreta) and retained placenta.

The incidence of mullerian duct anomalies varies widely. Septate uterus is a principal mullerian anomaly [1]. Uterine congenital abnormalities may include several gynecologic and obstetric problems. Obstetric problems are malpresentation, intrauterine growth restriction, retained placenta, and antepartum and postpartum hemorrhage [2].

Uterine atony has been an indication for the use of the B-Lynch procedure. However, B-Lynch suture is also useful in controlling hemorrhage in cases of placenta praevia and placenta accreta [3]. Several combinations of therapies are utilized for PPH, but no prior reports have described the techniques of external compression sutures with intrauterine gauze tampon in mullerian anomaly and placenta previa accreta.

## 2. Case Report

**Figure 1:**
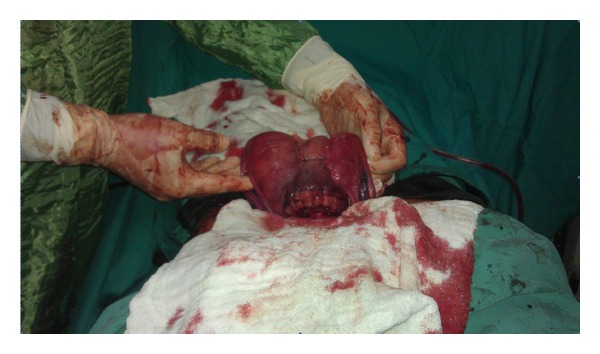


A 28-year-old woman, gravida 4, abortus 3, at 36-week gestation, was admitted to emergency service with vaginal bleeding and abdominal pain. A 35 weeks 4 days of gestation with normal amniotic fluid index and total placenta previa was examined in the abdominal ultrasound. In NST evaluation, uterine contraction with regular low amplitude was observed. Her vital signs were notable T/A: 120/70 mmHg, hearth rate was 84/min, body temparature was 36.8°C, and respiratory rate was 20/min. Laboratory tests were unremarkable. After getting the diagnosis of placenta previa, she was transferred to the operating room. A lower segment caesarean section was performed under general anaesthesia, with the delivery of an 2875 gr female and with an Apgar score of 8 and 9 at 1 and 5 minutes, respectively. Deep and thick uterine septum was detected in operation. The placenta was evaluated to extend across the cervical os, and controlled traction was unsuccessful. Manual evaluation of the placenta was found to invade the myometrium, and placenta was accreta. The uterine serosa layer was normal. After removal of placenta, a massive bleeding has occurred. Fundus massage was applied immediately. An infusion of 20 IU oxytocin 500 mL saline was given. Intramyometrial 0.2 mg/mL methylergonovine was performed. Routine medications failed to control hemorrhage. Therefore, additional surgical treatment was planned. Prior bleeding area was sutured with 1/0 vicryl. But the bleeding was not controlled. Therefore, 2/0 vicryl was prepared for B-Lynch suture. B-Lynch suture was performed, but the lower segment of suture was not closured. Ten gauze compresses were prepared. Both the gauze compresses were tied. Firstly, one end of the gauze compress was passed through the cervix. Secondly, lower segment of uterus was packed. The remaining gauze compresses were used to pack the fundus and cornu of the uterus. Then, incision line of uterus was stitched carefully. The B-Lynch suture was tied and patient was assessed for any vaginal bleeding by nurse (Figure 1). No vaginal bleeding was detected and abdominal incision was closed. Total 3000 mL saline and 80 IU oxytocin were infused on postoperative 24 hours. Total two units packed erythroctes were admitted (1 unit intraoperative and 1 unit postoperative). Eighteen hours after surgery, gauze compreses were removed vaginally in the operation room (Figure 2). The patient was discharged after two days. The patient was seen 1 week and 1 month later and no additional problem was detected.

## 3. Conclusion

PPH is an obstetric emergency that can occur following vaginal delivery or CS. The etiology of PPH is uterine atony, placental adhesion, retained placenta, retained clots, genital lesions or trauma, and disorders of coagulation. The incidence of postpartum haemorrhage has been estimated as 4 to 6% of all pregnancies [4, 5]. Management of PPH begins with conservative methods like bimanual uterine compression, use of uterotonics, uterine tamponade with balloons, and rarely arterial embolisation, the failure of which mandates surgical intervention. But combination of conservative therapies is usually necessary.

Hemostatic brace suture was described by B-Lynch in 1997. The B-Lynch suture has also been used successfully in combination with other methods such as balloon and uterine packing [6]. But especially uterine tamponade was achieved by packing the uterus with cotton gauze, a technique that had several disadvantages (uterine injury and infection). It requires general or regional anaesthesia.

Obstetrical complications have been reported to occur more commonly with mullerian duct anomalies, including increased risks of miscarriage, preterm delivery, intrauterine growth restriction, cervical incompetence, abnormal fetal presentation, pregnancy-associated hypertension, cesarean delivery, and antepartum and postpartum hemorrhage [7–10].

The suture has been also found to be useful in controlling bleeding in cases of placenta praevia and placenta accreta. It has been also used prophylactically in cases of morbidly adherent placenta, placenta praevia major, clotting factor deficiency, and so forth [11]. Uterine packing and B-Lynch suture are being used to arrest the bleeding from upper as well as lower uterine segment, where B-Lynch alone was thought to be ineffective.

We found that intrauterine gauze compress combined B-Lynch suture in this patient was highly effective in the management of PPH unresponsive to standard management. Intrauterine gauze compress combined B-Lynch brace suture is effective and a safe method in the patient with mullerian anomaly and placenta previa accreta. Combined use of intrauterine gauze compress and B-Lynch brace suturing results in direct compression of uterine walls in upper and placenta previa accreta segment. This combined effect helps in securing blood loss. Our results are comparable with another study [12]. This technic is simple to use and success is comparable to other methods (balloons). It can be used in uterine atony and placenta previa accreta. However, our study was a case report without a proper comparison group. We need randomised studies to compare its effectiveness with the other methods in the management of placenta previa accreta in mullerian anomaly in order to fully support its widespread use.

## Figures and Tables

**Figure 2:**